# Fertility loss and recovery dynamics after repeated heat stress across life stages in male *Drosophila melanogaster*: patterns and processes

**DOI:** 10.1098/rsos.241082

**Published:** 2024-10-02

**Authors:** Abhishek Meena, Alessio N. De Nardo, Komal Maggu, Sonja H. Sbilordo, Jeannine Roy, Rhonda R. Snook, Stefan Lüpold

**Affiliations:** ^1^ Department of Evolutionary Biology and Environmental Studies, University of Zurich, Zurich, Switzerland; ^2^ Department of Zoology, Stockholm University, Stockholm, Sweden

**Keywords:** development, *Drosophila melanogaster* Genetic Reference Panel, ectotherm, sperm viability, mechanism, temperature

## Abstract

Frequent and extreme temperatures associated with climate change pose a major threat to biodiversity, particularly for organisms whose metabolism is strictly linked to ambient temperatures. Many studies have explored thermal effects on survival, but heat-induced fertility loss is emerging as a greater threat to population persistence. However, while evidence is accumulating that both juvenile and adult stages heat exposure can impair fertility in their own ways, much less is known about the immediate and longer-term fitness consequences of repeated heat stress across life stages. To address this knowledge gap, we used male *Drosophila melanogaster* to investigate (i) the cumulative fitness effects of repeated heat stress across life stages, (ii) the potential of recovery from these heat exposures, and (iii) the underlying mechanisms. We found individual and combined effects of chronic juvenile and acute adult heat stress on male fitness traits. These effects tended to exacerbate over several days after brief heat exposure, indicating a substantial fertility loss for these short-lived organisms. Our findings highlight the cumulative and persistent effects of heat stress on fitness. Such combined effects could accelerate population declines, particularly in more vulnerable species, emphasizing the importance of considering reproduction and its recovery for more accurate models of species persistence.

## 1. Introduction

Climate change, particularly the rise of frequent and extreme heatwaves, poses a significant threat to global biodiversity [1–3]. Temperatures exceeding adaptive thermal limits can harm organisms [4–7], leading to population declines, extinctions or range shifts across ecosystems [8,9]. Consequently, thermal tolerance limits are widely studied and used to predict the global risk of biodiversity loss [10]. However, the persistence of populations or species also depends on successful reproduction, which can be disrupted well below the lethal temperature [6,7,11]. Integrating thermal fertility limits into species distribution and persistence models could thus significantly improve their accuracy [7,12]. Sublethal effects on reproduction can also have more profound evolutionary consequences than mortality alone [6,7], for example by being passed down through transgenerational effects [13,14]. Understanding how the reproductive performance of organisms responds to and recovers from heat stress is thus crucial.

While endothermic organisms like birds and mammals can regulate their body temperature to some extent [15,16], even they can suffer reproductive disruption from heat stress [17]. This can manifest as impaired sperm production [18–20], leading to low sperm concentration [21,22] or even complete absence of sperm [23,24]. Additionally, heat stress can cause higher proportions of morphologically abnormal or poorly motile sperm [19,25,26].

The reproductive function of ectotherms (e.g. invertebrates) is often even more vulnerable to high temperatures because their physiology, metabolism and behaviour are intimately linked to their ambient temperature [27–30]. Ectotherms often experience infertility well below their lethal limit [7,11]. This vulnerability is further amplified during specific life stages [31–33]. For example, adult stages in holometabolous insects are often more thermally sensitive than earlier life stages [31,32,34]. This increased sensitivity in adult stages partly stems from a reduced capacity to repair damage or to modify morphology and physiology as juveniles can during metamorphosis [35,36]. Juveniles can plastically alter their growth rate [37], final body size [38], investments in reproductive tissue [39] or the metabolic rate [30,36] to maintain homeostasis and avoid fitness consequences under unfavourable conditions. While some of these consequences are permanent (e.g. body size), others may be partially reversible (e.g. metabolic rate or reproductive fitness [32,39]).

Sublethal fertility loss can be the result of thermal damage to germlines and reproductive physiology [6,17,40,41]. In general, spermatogenesis appears more thermally sensitive than oogenesis [14,42,43], rendering male fertility an important parameter for determining reproductive capacity and fitness of populations under changing climates. Since gonad formation and spermatogenesis often begin during larval development [44], chronic heat exposure during this phase could harm the developing reproductive tissue [39] or disrupt spermatogenic processes, resulting in reduced sperm quantity or quality, and consequently fertility, later in life [11,45,46]. However, the extent to which such fitness loss is temporary or permanent remains poorly studied. This is because most research to date has measured male fertility immediately after eclosion following development under constant or fluctuating heat stress, without tracking longer-term effects [43,47,48]. Only few studies have explored potential recovery from thermal stress [14,31,32,39,45,49]. Those studies that have indicate that fertility may at least partially recover over the first few days after emergence, but the recovery period tends to increase with temperature [32,39,45,50].

Heat stress in adult individuals can also impact their fertility. The consequences could be relatively short-lived if a heatwave primarily affects mature sperm, as these can be replaced by new ones relatively soon. By contrast, damage to the reproductive tissue or spermatogenesis could impair a male’s fertility over a longer period. Similar to juvenile heat stress, little is known about the recovery of fertility after heat exposure. One study of the flour beetle *Tribolium castaneum* has shown that offspring production can recover completely within about three weeks of a 5-day heatwave [32]. This result indicates that some effects of heat stress are reversible [51,52], yet an extended recovery period can still have considerable fitness consequences.

Despite a growing body of literature on thermal fertility effects [53], four key knowledge gaps remain. First, most studies have examined heat stress during either the juvenile [11,39,43] or the adult stage [14,54], hindering direct comparisons of fertility consequences between life stages [53]. Second, studies that did compare stage-specific thermal effects typically exposed individuals to a single heat treatment, limiting our knowledge of how individuals respond to repeated heat bouts across stages [14,31,32,34,54,55]. For example, it is unclear whether juvenile heat stress increases the adult thermal tolerance through heat hardening [56] or if repeated heat stress has cumulative detrimental effects on reproductive function [14]. Third, we know little about the potential of recovery after heat exposure in general, and even less so in the context of life stage-specific exposure or repeated bouts. Finally, the mechanisms underlying heat-related fertility loss remain elusive, particularly measured in an integrated way.

To examine how male reproductive function responds to heat stress across life stages, we used *Drosophila melanogaster* isogenic lines from the *Drosophila melanogaster* Genetic Reference Panel (DGRP [57]) with known variation in heat tolerance during development [43,47,48]. We reared all larvae at either 24.5 or 28°C, reflecting optimal or stressful temperatures, respectively [11]. Upon adult eclosion, we exposed the males of each line to a 4 h temperature treatment at 24.5°C (control), 28°C (thermal stress) or 36.5°C (severe stress) and then measured male fertility traits 1 and 5 days after the heat stress. We addressed the following questions: (i) How does repeated heat stress across life stages affect male reproduction? (ii) How does life stage-specific and repeated heat stress affect the males’ ability to recover? (iii) What are the mechanisms underlying the reproductive consequences of heat stress in males? By investigating these questions, we aimed to improve our understanding of how heat stress impacts male fertility across life stages and recovery periods. Such knowledge is crucial for predicting the population-level effects of climate change on insect biodiversity and ecosystem function.

## 2. Material and methods

### 2.1. Study organisms

We used isogenic lines of the DGRP. These lines were generated by 20 generations of full-sib inbreeding from a single population in Raleigh, North Carolina, where summers are hot and humid with average high temperatures ranging between 21 and 32°C (https://weatherspark.com/). The advantage of using isogenic lines is that each genotype can be exposed to different treatments simultaneously, which can simulate an individual’s response to different conditions in a replicated manner.

The thermal reaction norms of male fertility in response to different constant developmental temperatures (25, 27 and 29°C) have been described for 127 DGRP lines [43]. Follow-up studies have used a subset of 30 of these lines based on the thermal tolerance of male fertility [47,48]. Fifteen of these lines tolerated high developmental temperatures (29°C) with minimal fertility loss (hereafter ‘high’ tolerance), while the remaining 15 lines were heat-sensitive at 29°C (hereafter ‘low’ tolerance) [43,47,48]. Throughout the experiment, we used the same isolines for focal males, along with standardized wild-type females (strain Canton special, CS [47,48]). However, as one low-tolerance line did not produce enough eggs to distribute in adequate numbers across all treatments, we were able to only use 29 lines.

All males and females were maintained on cornmeal medium (75 g glucose, 100 g fresh yeast, 55 g corn, 10 g flour and 15 ml nipagin antimicrobial agent per litre food media) under standardized laboratory conditions (24.5 ± 0.5°C, 60–70% relative humidity and 14L : 10D photoperiod).

### 2.2. Temperature effects on male fertility and its recovery

**Figure 1.**
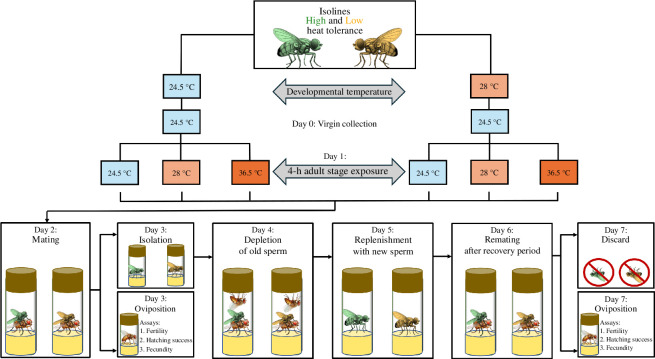
Schematic representation of the experimental design for reproductive fitness assays using 15 high- and 14 low-tolerance isolines.

For each DGRP line, we allowed adults to oviposit on grape juice-agar medium (300 ml water, 670 ml grape juice, 22 g agar, 20 g sugar, 19 g yeast, 20 ml nipagin per litre) for 15–18 h and then transferred their eggs to culture vials with standard fly media (50 eggs on 6 ml food). Half of the vials per line developed at a constant 24.5°C (near the optimal temperature), the other half at a constant 28°C (thermal stress). The common females (CS) were reared at 24.5°C in bottles with 200 eggs on 100 ml food. We collected experimental males and standard females within 7 h of emergence to ensure virginity and kept them separated by sex at 24.5°C for 1 day. We collected focal males using an aspirator to avoid potential behavioural effects of ice or CO_2_ anaesthesia. One day after emergence, we subjected males to an adult heat treatment by transferring them in groups of 10 to glass vials with agar medium (to prevent dehydration) and placing them into a water bath at 24.5, 28.0 or 36.5°C for 4 h, resulting in *n* = 2 vials × 10 males × 29 isolines × 2 developmental treatments × 3 adult treatments = 3480 males (figure 1). We chose a slightly lower juvenile heat-stress temperature than the 29°C used in previous studies on the same fly population [43,48], because the near-complete fertility loss in many low-tolerance genotypes after developing at 29°C would have prevented studying any additional effects of adult heat stress. By contrast, the adult heat stress temperature of 36.5°C was near the fertility limit for adult flies based on our pilot study. We used the 28°C treatment at the adult stage to compare the effect of identical larval and adult temperatures more directly and to address questions about acclimatization. Immediately after the adult treatment, we transferred males to vials with standard fly food.

The day after the adult thermal treatment, we paired each male with a standard virgin female (3–4 days old) for 24 h in a glass vial with standard fly food. Then, we transferred each female to an oviposition vial with grape juice-agar medium for another 24 h before estimating three male fitness traits (figure 1): (i) fertility as the presence of any hatched larvae (greater than or equal to 1 = fertile, 0 = infertile), (ii) the proportion of eggs hatched (fertilization success), and (iii) male-mediated female fecundity (total number of larvae).

On the 4th day after the heatwave, we provided each male with two non-experimental virgin females for a day to substantially deplete old sperm in storage [58], followed by a day to recover in isolation. Five days after the heat stress, we repeated the fitness assays as described above to assess if males had recovered from their heat stress and regained full reproductive function (figure 1). Since spermiation (post-meiotic sperm development) lasts 5 days in *D. melanogaster* [44], sperm transferred at this stage should have matured after the heat stress, whereas those transferred in the first assay were mature during exposure. All sperm were exposed to the developmental temperature during their mitotic and meiotic cell divisions (also approximately 5 days; [44]), but these stages are far less sensitive than the late spermatogenic stages [31].

### 2.3. Assessment of male reproductive organs

To quantify in more detail the size of reproductive organs (accessory gland size, seminal vesicle area as a proxy of mature sperm reserves, and the presence of sperm in it) in response to different temperature treatments. To this end, we reared a randomly selected subset of lines (five high and five low) and reared each at 24.5 and 28°C, respectively. After emergence, we subjected males from each line and treatment to 4 h of adult thermal treatment at 24.5 or 36.5°C in a full-factorial design. These thermal treatments essentially followed the same general protocol as in the fitness assay, except for omitting the 28°C adult heat pulse due to relatively little difference to the 24°C adult treatment among the fitness traits. We then froze 10–15 naive males from each line and treatment at each of two time points: 1 and 5 days after the adult temperature treatment, respectively. For the latter time point, we additionally froze sets of males that had been paired with two virgin females for 24 h on the 3rd day, followed by a day to replenish their sperm reserves. Later, we measured the thorax length of all males using a reticular eye piece mounted on a Leica stereomicroscope, dissected them in 1% PBS and photographed them at 32× magnification under the same stereomicroscope. We then checked for the presence/absence of sperm in the seminal vesicle and measured both the accessory gland and seminal vesicle areas for all males using ImageJ v. 1.54i.

### 2.4. Sperm viability

Of the same cohorts as for reproductive organs, we dissected four–five males from each line and treatment at the same two time points. For the assay, we anaesthetized these males on ice and immediately extracted their reproductive tract in 5 µl Beadle solution (128.3  mM NaCl, 4.7  mM KCl, 2.3  mM CaCl_2_) on a microscope slide. After piercing one seminal vesicle using an insect pin, we released sperm by gentle pressing, added 5 µl LIVE/DEAD sperm viability stain (Molecular Probes L-7011) following the protocol outlined in Tourmente *et al*. [59] and, after 5 min of incubation, counted the live (green) and dead (red) sperm using ImageJ v. 1.54i to calculate the proportion of live sperm [59].

### 2.5. Statistical analyses

We conducted all analyses in R v. 4.3.2 [60], using (generalized) linear mixed-effects models (G)LMMs, with the line-specific thermal tolerance category (high, low [43,47,48]), developmental temperature, adult temperature and their interactions as explanatory variables. We further controlled for experimental blocks and non-independence of males within isolines as random effects. In cases of overdispersion, we additionally included an observation-level random effect.

For each trait, we first conducted a full model including the four-way interaction between the two stages of heat exposure, the line-specific heat tolerance, and the time point of assessment (i.e. 1 or 5 days after the adult treatment). However, since four-way interactions are difficult to interpret, and in some cases, different data distributions between sampling days or limited statistical power made it difficult to satisfy all model assumptions, we used these models primarily to assess if the general patterns were different between these time points. For more detailed interpretation and visual representation of the results, we then conducted separate analyses for each of the two post-treatment time points.

We analysed models on fertility, sperm presence in the seminal vesicles and sperm viability using *glmer* (*lme4* package) [61] with a binomial distribution. Since fecundity and fertilization were zero-inflated, we analysed these using the *glmmTMB* package [62] with a negative binomial and beta-binomial error distribution, respectively. For seminal vesicle and accessory gland areas, we used linear mixed models as implemented in *lmerTest* [63].

To balance model simplicity and explanatory power, we sequentially removed non-significant interaction terms (*p* > 0.1), but always retained all focal main effects due to their biological relevance in our study. Specifically, we first removed any non-statistically significant three-way interaction and then sequentially non-significant two-way interactions in the order of decreasing *p*-values. In each simplification step, we compared the models with and without the focal interaction using a likelihood ratio test. We assessed model performance (residual diagnostics, evaluations of overdispersion, zero inflation, or outliers) using the *DHARMa* [64] and *car* packages [65] for GLMMs and LMMs, respectively. We visualized our models using the *sjPlot* package [66].

## 3. Results

### 3.1. Male fertility

**Figure 2.**
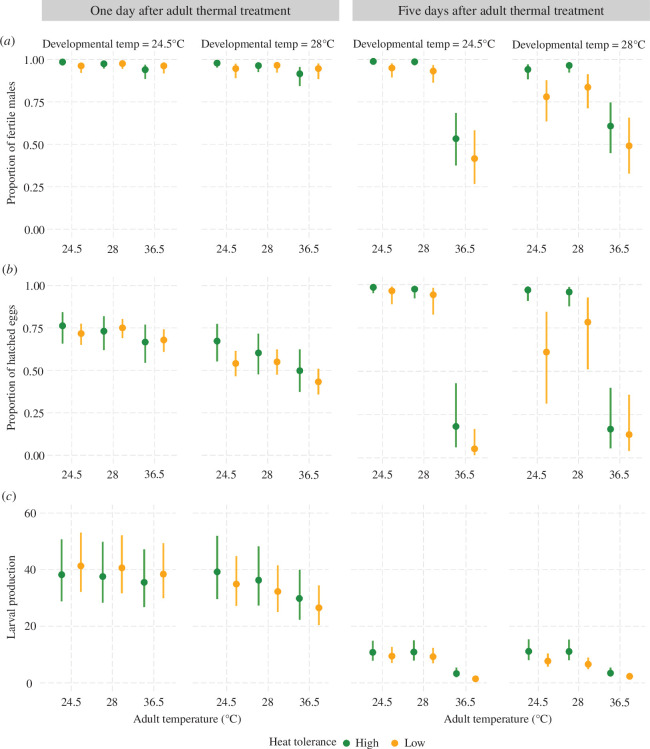
Effects of juvenile and adult heat stress on (*a*) the proportion of fertile males, (*b*) the proportion of hatched eggs and (*c*) larval production. The left panels show the results of 1 day after adult thermal treatment, and those on the right represent the results of 5 days after thermal treatment. The green and golden markers represent the genotypes with high and low thermal tolerance, respectively. Means with 95% confidence intervals are shown.

In a binomial GLMM across *n* = 3742 observations of 2335 males from all 29 isolines, for which their mated female laid at least one egg, we found a trend for a four-way interaction (*χ* ^2^ = 4.875, d.f. = 2, *p* = 0.087; electronic supplementary material, table S1), suggesting that the thermal effects on male fertility tended to differ between 1 and 5 days after the adult heat stress. However, not all males had data for both days. To better interpret the complex interaction, we conducted separate analyses for either sampling day. On the day after the adult treatment (*n* = 1859 males), we found adult heat stress to reduce the probability of producing at least one larva (*χ* ^2^ = 10.962, d.f. = 2, *p* = 0.004), with a trend in the same direction for the developmental temperature (*χ* ^2^ = 2.794, d.f. = 1, *p* = 0.095; figure 2*a*, electronic supplementary material, table S2). A significant interaction between thermal tolerance category and adult temperature (*χ* ^2^ = 7.126, d.f. = 2, *p* = 0.028) indicated that the isolines characterized by high thermal tolerance during development [43,48] showed a steeper decline across adult temperatures than the more heat-sensitive isolines. Hence, developmental heat tolerance does not necessarily transcend life stages.

Following a 5-day recovery period across *n* = 1883 males, both developmental (*χ* ^2^ = 6.548, d.f. = 1, *p* = 0.010) and adult heat stress (*χ* ^2^ = 253.275, d.f. = 2, *p* < 0.001) negatively impacted fertility, with a significant interaction between these life stages (*χ* ^2^ = 29.815, d.f. = 2, *p *< 0.001). Specifically, the adult heat stress at 36.5°C dramatically decreased male fertility after this period, suggesting no recovery. In contrast to 1 day post-stress, the isolines with low thermal tolerance now exhibited a stronger negative effect of adult temperature than their more tolerant counterparts (*χ* ^2^ = 12.611, d.f. = 2, *p* = 0.002; figure 2*a*; electronic supplementary material, table S3).

### 3.2. Hatching success

For a more refined estimate of male fertilizing capacity than the presence/absence of any hatched egg, we further quantified the proportion of hatched eggs laid over a 24 h period as a proxy of fertilization success. Restricting our analyses to those 3330 males with at least five eggs from which to estimate hatching success, there was no significant four-way interaction (χ^2^ = 1.889, d.f. = 2, *p* = 0.389; electronic supplementary material, table S4). Yet, for consistency with the other analyses and because the data at the two time points required different error distributions in the models, we again conducted separate analyses between the days post-adult treatment. After 1 day (*n* = 1595), there was a significant decline in hatching success with increasing developmental (beta-binomial GLMM: χ^2^ = 457.194, d.f. = 1, *p* < 0.001) and adult temperature (*χ* ^2^ = 141.769, d.f. = 2, *p* < 0.001), with an interaction between life stages (χ^2^ = 10.841, d.f. = 2, *p* = 0.004). Specifically, hatching success declined more steeply with increasing adult temperature following juvenile heat stress. While the reduction in hatching success was stronger in low- compared with high-tolerance lines in response to developmental heat stress (*χ* ^2^ = 22.709, d.f. = 1, *p *< 0.001), high-tolerance lines were more severely affected by adult heat stress (*χ* ^2^ = 23.361, d.f. = 2, *p *< 0.001; figure 2*b*, electronic supplementary material, table S5).

Five days after the adult heat treatment, there was no significant interaction between developmental and adult temperature (beta-binomial GLMM: *χ* ^2^ = 0.200, d.f. = 2, *p* = 0.905, *n* = 1735; figure 2*b*, electronic supplementary material, table S6). However, both the developmental (χ^2^ = 22.434, d.f. = 1, *p* < 0.001) and adult temperatures had a negative main effect (*χ* ^2^ = 173.298, d.f. = 2, *p* < 0.001), and each interacted with the heat tolerance (developmental: *χ* ^2^ = 5.660, d.f. = 1, *p* = 0.017; adult: *χ* ^2^ = 6.524, d.f. = 2, *p* = 0.038). Hence, we found a stronger negative response of low-tolerance lines to developmental heat stress compared with the barely affected high-tolerance lines, whereas the latter showed a more substantial drop between 28 and 36.5°C adult temperature. Overall, compared with the steady decline in hatching success with increasing adult temperature 1 day after the treatment, here we primarily found a drop in the 36.5°C treatment.

### 3.3. Male-mediated female fecundity

Since a male’s fitness ultimately does not only depend on the proportion of fertilized eggs but on the number of larvae produced, we next tested how heat stress affected male mediation of female offspring production (e.g. via transfer of fecundity-enhancing seminal fluid proteins and functional sperm) in a non-competitive context. A GLMM with a negative binomial error distribution revealed a significant four-way interaction between developmental and adult temperatures, male heat tolerance and the day post-adult treatment (*χ* ^2^ = 74.793, d.f. = 2, *p* < 0.001, *n* = 3742; electronic supplementary material, table S7). Hence, the effects of thermal stress on high- versus low-tolerance lines differed between time points after adult heat stress. To better interpret these results, we again conducted separate models for each day. For the first time point, larval production was reduced by both developmental (*χ* ^2^ = 13.411, d.f. = 1, *p* < 0.001, *n* = 1859) and adult temperature (*χ* ^2^ = 14.682, d.f. = 2, *p* = 0.001). An interaction between these two predictors further suggested a greater reduction in male-mediated fecundity in response to adult compared with developmental temperature (*χ* ^2^ = 6.018, d.f. = 2, *p* = 0.049). Additionally, an interaction between thermal tolerance and developmental temperature indicated a stronger negative heat stress effect on low- compared with high-tolerance lines (*χ* ^2^ = 9.027, d.f. = 1, *p* = 0.003; figure 2*c*, electronic supplementary material, table S8).

Following 5 days of recovery, we obtained broadly similar results, but with a steeper drop in fecundity after an adult exposure to 36.5°C (*χ* ^2^ = 136.599, d.f. = 2, *p* < 0.001, *n* = 1883) and a weaker effect of developmental temperature (*χ* ^2^ = 3.328, d.f. = 1, *p* = 0.068). Overall, the low-tolerance lines produced significantly fewer offspring than the high-tolerance ones (*χ* ^2^ = 8.416, d.f. = 1, *p* = 0.004; figure 2*c*, electronic supplementary material, table S9). The weak three-way interaction, albeit not statistically significant (*χ* ^2^ = 5.410, d.f. = 2, *p* = 0.067), further suggested that the low-tolerance lines tended to be more strongly affected by developmental heat stress, resulting in a considerable fitness loss even if kept around the optimal temperature after eclosion.

### 3.4. Sperm presence

**Figure 3.**
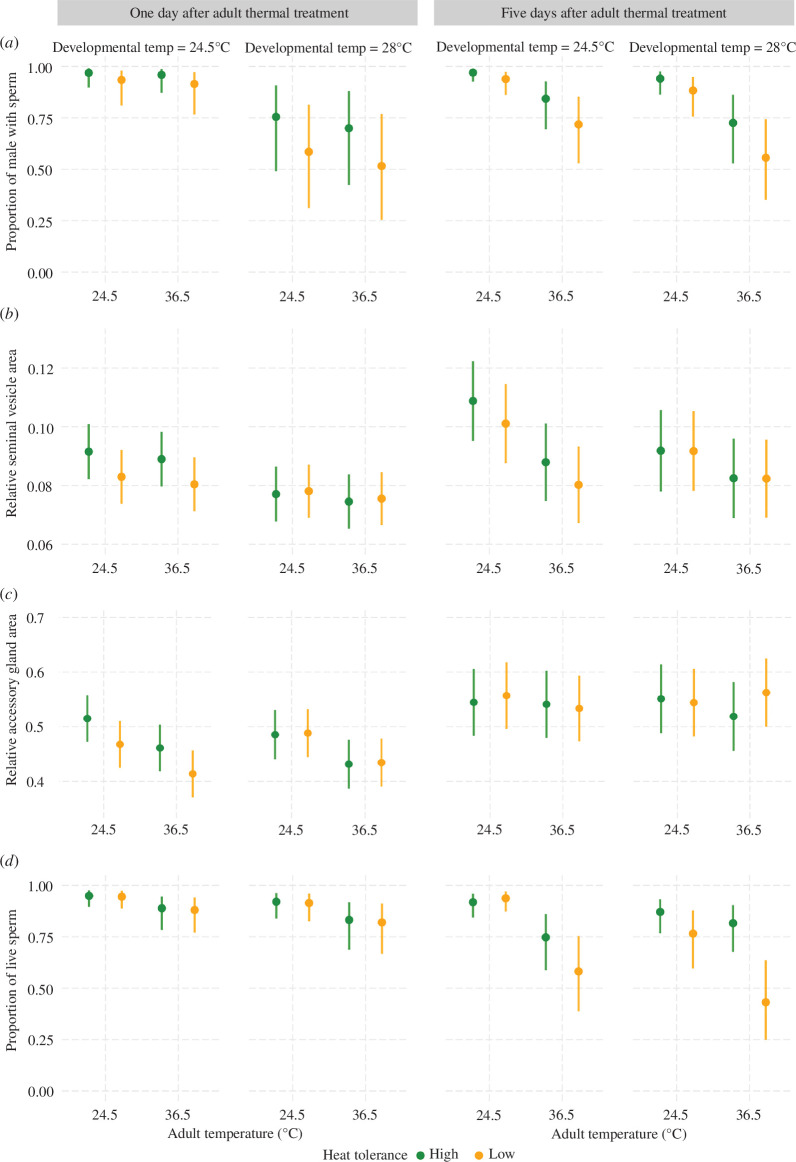
Effects of juvenile and adult heat stress on (*a*) the proportion of males with sperm, (*b*) the relative seminal vesicle area, (*c*) the relative accessory gland area and (*d*) the proportion of live sperm. The left panels show results of 1 day after adult thermal treatment, and the right panels reflect the results of 5 days after the thermal treatment. The green and golden markers represent the genotypes with high and low thermal tolerance, respectively. Means with 95% confidence intervals are shown.

Given the detrimental effects of developmental and adult heat stress on male fitness, we next examined potential mechanisms underlying the observed fitness loss. First, we tested if the decline in fertility and fecundity resulted from impaired sperm production by quantifying the proportion of males that had any sperm stored in their seminal vesicles. In a binomial GLMM across *n* = 751 males from 10 isolines, we found no four-way interaction (*β* = −0.790 ± 2.106, *Z* = −0.375, *p* = 0.708), but day interacted with both developmental and adult temperature (both |*Z| *≥ 0.886, *p *< 0.001; electronic supplementary material, table S10). Within each day, however, there were no significant interactive effects, reducing our models to main effects only. For those males dissected the day after the adult thermal treatment, we found high developmental temperature to significantly lower the probability of mature sperm being present in the seminal vesicles after eclosion (*n* = 415, *β* = −2.313 ± 0.352, *Z* = −6.580, *p *< 0.001; figure 3*a*, electronic supplementary material, table S11). Unsurprisingly, adult temperature did not affect this probability (*β* = −0.280 ± 0.293, *Z* = −0.954, *p* = 0.340), since sperm may have already been present at that time [31]. The thermal tolerance of the isolines also had no effect (*β* = −0.782 ± 0.786, *Z* = −0.995, *p* = 0.320), although this negative result could, at least in part, be attributable to many fewer isolines being used than for the fitness assays.

These results changed after males had the opportunity to deplete and renew their sperm reserves for 5 days after the adult temperature treatment. Now, the probability of mature sperm being present in the seminal vesicles declined in response to adult heat stress (*n* = 336, *β* = −2.137 ± 0.315, *Z* = −6.780, *p* < 0.001), with a negative trend for developmental heat stress (*β* = −0.503 ± 0.284, *Z* = −1.770, *p* = 0.076) and no effect of thermal tolerance (*β* = −0.747 ± 0.582, *Z* = −1.282, *p* = 0.200; figure 3*a*, electronic supplementary material, table S12). Hence, this post-treatment period was not sufficient for many heat-stressed males to produce and release a new cohort of sperm, thereby probably explaining why a stronger effect was found at this stage in all our initial fitness assays.

### 3.5. Relative seminal vesicle area

As the presence and absence of sperm in the seminal vesicles is a relatively coarse index of a male’s fertilization capacity, we additionally used the mean area of both seminal vesicles as a proxy for the number of sperm stored [39]. In an LMM controlling for male body size (thorax length) and the presence/absence of any sperm (both *t *≥ 3.826, *p *< 0.001), there was no significant four-way interaction on seminal vesicle size (*β* = −0.007 ± 0.009, *t* = −0.576, *p* = 0.450), but day contributed to several interactions (electronic supplementary material, table S13). Considering only the day after the adult treatment, seminal vesicles, again controlling for male size and sperm presence (both *t *≥ 2.893, d.f. = 1, *p* ≤ 0.004), were significantly smaller after developmental heat stress (*n* = 415, *β* = −0.014 ± 0.002, *t* = −5.971, *p* < 0.001). Adult heat stress showed a similar trend, though non-significant, effect (*β* = −0.003 ± 0.001, *t* = −1.880, *p* = 0.061; figure 3*b*, electronic supplementary material, table S14). Furthermore, there was a significant interaction between development temperature and thermal tolerance (*β* = 0.010 ± 0.003, *t* = 3.457, *p* = 0.001), with high-tolerance lines having larger seminal vesicles than the low-tolerance lines following development at 24.5°C, but essentially the same reduced size as the latter under stressful developmental conditions.

After the 5-day recovery period, the seminal vesicles, accounting for thorax length and sperm presence (both *t* ≥ 2.804, *p* ≤ 0.005), were still smaller under high developmental (*n* = 336, *β* = −0.017 ± 0.003, *t* = −4.877, *p* < 0.001) and adult temperature (*β* = −0.021 ± 0.003, *t* = −8.133, *p* < 0.001). These two temperature treatments further had an interactive effect (*β* = 0.011 ± 0.004, *t* = 3.140, *p* = 0.002; figure 3*b*, electronic supplementary material, table S15), indicating that 36.5°C post-eclosion reduced the seminal vesicles to a similar size, but that vesicles derived from the 28°C developmental treatment were already smaller from the beginning. The additional interaction between development temperature and thermal tolerance (*β* = 0.008 ± 0.004, *t* = 2.054, *p* = 0.041) again suggested that high-tolerance lines grew larger seminal vesicles under benign conditions than any other treatment group, but that heat stress reduced these organs to a similar size as in the low-tolerance lines.

### 3.6. Relative accessory gland area

In an LMM controlling for thorax length (*n* = 751, *β* = 0.099 ± 0.027, *t* = 3.726, *p* < 0.001), there were no higher-order effects, but day interacted with both developmental and adult heat treatments (both *t* ≥ 1.101, *p* < 0.001; electronic supplementary material, table S16). For males measured on the day after the adult treatment, the accessory gland area was negatively affected by adult temperature (*n* = 415, *β* = −0.054 ± 0.008, *t* = −7.186, *p* < 0.001) and an interaction between developmental temperature and thermal tolerance (*β* = 0.050 ± 0.015, *t* = 3.257, *p* = 0.001; figure 3*c*, electronic supplementary material, table S17), again controlling for thorax length (*β* = 0.083 ± 0.034, *t* = 2.439, *p* = 0.015). This interaction suggests relatively smaller accessory glands for low-tolerance lines than their high-tolerance counterparts at 24.5°C, but similar sizes between these groups at 28°C.

After 5 days of recovery, again controlling for thorax length (*n* = 336, *β* = 0.123 ± 0.039, *t* = 3.147, *p* = 0.002), we found a weak three-way interactive effect on accessory gland area between thermal tolerance and the developmental and adult temperatures (*χ* ^2^ = 3.851, d.f. = 1, *p* = 0.050; figure 3*c*, electronic supplementary material, table S18). Specifically, high-tolerance lines exhibited slightly smaller and low-tolerance lines slightly larger accessory glands after adult heat stress than under benign conditions, but only if they had already been exposed to elevated temperature during development. None of the remaining main or interactive effects were statistically significant (all *χ* ^2 ^≤ 1.345, d.f. = 1, *p *≥ 0.246).

### 3.7. Sperm viability

Finally, we examined the proportion of viable sperm among those produced by males either shortly after the adult heat treatment or as a new cohort after a recovery period. In a binomial GLMM across *n* = 182 males from 10 isolines, the four-way interaction was not statistically significant (*β* = 1.937 ± 1.659, *Z* = 1.168, *p* = 0.243), but day again contributed to several interactions (though some marginally non-significant; electronic supplementary material, table S19). In fact, separate analyses revealed different patterns between sampling days. One day after the adult thermal treatment, adult temperature had a significant negative impact on sperm viability (*β* = −0.850 ± 0.285, *Z* = −2.983, *p* = 0.003; figure 3*d*, electronic supplementary material, table S20), and developmental temperature showed a similar, though non-statistically significant, trend (*β* = −0.477 ± 0.288, *Z* = −1.657, *p* = 0.097). Thermal tolerance had no effect (*β* = −0.084 ± 0.351, *Z* = −0.239, *p* = 0.811).

After 5 days of recovery, both high developmental and adult temperature lowered sperm viability (*n* = 82, both *z* ≤ −2.951, d.f. = 1, *p* < 0.001), with an interaction between them suggesting a cumulative impact (*β* = 0.918 ± 0.213, *Z* = 4.316, *p* < 0.001). Both temperature treatments further interacted with the thermal tolerance of the isolines (developmental: *β* = −1.011 ± 0.220, *Z* = −4.592, *p* < 0.001; adult: *β* = −1.040 ± 0.224, *Z* = −4.653, *p* < 0.001; figure 3*d*, electronic supplementary material, table S21), both showing a stronger decline in the low- compared with high-tolerance lines. These findings may at least partially explain the observed decline in our fitness assays.

## 4. Discussion

Thermal effects on the reproductive function of organisms, particularly heat stress in ectotherms, have attracted considerable attention in recent years [53]. However, in contrast to a wealth of studies on the fitness consequences of elevated temperatures during either developmental [11,39,43,48] or adult stages [7,14,54], we still know little about the effects of multiple heat exposures across life stages, the permanency of these effects, or the underlying mechanisms. Here, we addressed these knowledge gaps by investigating how sublethal heat stress at different life stages affects male reproductive performance in *D. melanogaster*, both immediately and several days after exposure. We found that heat stress during both developmental (28°C) and adult stages (36.5°C) impairs male fertilizing capacity and offspring production. These effects were often exacerbated over the 5-day recovery period, indicating long-lasting consequences for male reproductive health.

One mechanism for this decline in reproductive performance is likely to be disrupted sperm production. We observed a lack of mature sperm in the seminal vesicles after heat stress, suggesting impaired spermiation and reduced sperm maturation [31,46,67,68]. Additionally, Kurhanewicz *et al*. [68] highlighted that spermatocytes have a limited tolerance to heat stress due to their inability to suppress mobile DNA elements, leading to DNA damage and genomic alterations that in turn decrease their fertilization capacity. These findings align with our results, which show decreased fertilization success following heat exposure [14,69]. Furthermore, heat stress not only reduced fertility or fertilization success but also negatively affected the total offspring number. This reduction can probably be attributed to the combined effects of reduced sperm quality and quantity, as evidenced by relatively smaller seminal vesicles and lower sperm viability, consistent with prior findings [31,39,46,67]. Overall, our results suggest that heat stress across life stages can impair both sperm quality and quantity, leading to decreased fertilization capacity and, ultimately, lower offspring production.

Our findings are consistent with previous studies showing that increasing heat stress reduces reproductive performance [11,14,32,39,43,46,48], potentially leading to temporary or permanent sterility [11,46,50,67]. Gandara & Drummond-Barbosa [67] identified a potential mechanism for this decline, attributing the drastic fertility drop to both low sperm abundance and poor quality following chronic adult exposure to warm temperatures. This reduction was not due to a decline in germline stem cell or spermatid numbers, but rather suggested heat-induced elimination of sperm via an apoptosis-independent mechanism during the last stage of spermatogenesis [67]. This aligns with our observations of reduced sperm quantity and viability following heat stress. Additionally, heat stress could induce defects in spermatogenesis [31,67], DNA damage [68], abnormal cyst elongation [46,67], incomplete sperm maturation and migration [67], or reduced sperm motility [70]. All these factors could contribute to reduced fertilization efficiency [71] or capacity to support embryogenesis [67], ultimately leading to decreased reproductive output [11,31,32,67].

Our analyses revealed that heat stress (36.5°C) for 4 h at adult stages has a stronger negative fitness effect than chronic heat stress (28°C) of juveniles, particularly after a 5-day recovery period. The difference between life stages may at least partly be explained by the capacity for physiological and metabolic repair and restructuring in juveniles, particularly during metamorphosis [34,35,72]. In contrast, damage incurred during the adult stage might directly affect both gametes and gonads, with more limited opportunities for repair [14,31,32]. This underscores the differential vulnerability of males to heat stress across life stages. Similar trends of increased heat sensitivity of reproductive function towards adult males have been reported in other studies, such as the diamondback moth *Plutella xylostella* [73] or *D. melanogaster* [31]. An alternative, more methodological explanation for this stage-specific effect, however, is that the adult stress temperature was close to the thermal fertility limit whereas the developmental stress was 1°C below the temperature inducing near-complete reproductive failure in the low-tolerance lines in previous studies [43,48]. It is thus possible that our treatment did not capture the full impact on juveniles compared with our adult treatment. Zwoinska *et al*. [43] reported a significant decline in fertility between 27 and 29°C. Our weaker effects at 28°C would suggest a much narrower window, with fertility dropping primarily between 28 and 29°C. If so, such a sharp shift in reproductive function could create intense selection for adaptations that increase heat tolerance compared with a more gradual change. A threshold-like loss of function also has implications for projections of population shifts, in that a small temperature change around the critical point could be highly consequential.

The effects of heat stress can be temporary, with individuals regaining their reproductive competence over time [11,31,32,39,45,50]. Recovery time depends on various factors, including the intensity [45,50], frequency [14,32] and duration of thermal stress [34], as well as the trait [50], species-specific tolerance [7,74] and life stage [31,32,34,75,76]. For instance in *D. melanogaster*, exposure to elevated temperatures during development significantly impairs spermatogenesis, leading to abnormal sperm production, delayed maturation, reduced organ size, higher spermatid mortality or incomplete spermatid elongation over a 10-day post-exposure period (encompassing a full spermatogenic cycle [11,39,46]). Similarly, double heat exposures within the same life stage (adult) of male *T. castaneum* have additive effects on testicular function and spermatogenesis, reducing sperm abundance and quality that ultimately lead to lower fertilization success and offspring production than single heat exposures [14,32]. Furthermore, heat exposure across life stages can lead to a reduction in total fecundity [73,77]. With increasing magnitude and frequency of heatwaves within a life stage, the recovery period can extend substantially [32,45,67].

Heat shock proteins (HSPs), activation of heat shock transcription factors, modulation of biogenic amines and the expression of other genes are known to play a critical role in recovery and protection from heat exposure [78–80]. These mechanisms assist in protein protection, repair and replacement of gametes damaged by heat stress, and in promoting spermatogenesis, a continuous process throughout the reproductive lifespan [81]. However, despite these mechanisms, our findings indicate that heat stress can cause a substantial reduction in male reproductive function. Furthermore, in our study, the combination of developmental and adult sublethal heat stress had a more pronounced impact, particularly by 5 days after the last heat exposure, suggesting that the recovery period probably extends beyond that caused by stress during a single life stage. Longer recovery periods after multiple heat exposures have also been reported in *T. castaneum* [32] and *D. virilis* [56]. Given the short lifespan of *D. melanogaster*, even a 5-day period of reduced fertility or fecundity can have a significant impact on lifetime reproductive success. Additionally, we did not observe any evidence for hardening between life stages, which contrasts with Jørgensen *et al*.’s [50] study on *D. buzzatii,* in which hardening buffered the effects of heat stress on male reproductive traits and facilitated faster recovery. These differences in outcomes could either reflect different responses between species or be the result of methodological differences. For example, Jørgensen *et al*. [50] exposed their flies to more than one heat stress within the same life stage, which might render heat hardening more effective than heat stress at different life stages, as in our study. Further research on the combined effects of multiple heat bouts within and between distinct life stages might help to reveal the potential of hardening in protecting against fitness loss.

Our observations suggest that genotypes with low thermal tolerance suffered more severe consequences and slightly slower recovery in most of the traits compared with those with high thermal tolerance, highlighting important genetic variation [43,46,48]. However, the genetic variation observed in this study was not as pronounced as reported previously [43,47,48]. This discrepancy could be due to our developmental temperature of 28°C compared with 29°C in these previous studies. Here, we chose this temperature slightly below the critical fertility limit as exposing juveniles to temperatures near reproductive failure would have prevented the detection of additional effects at the adult stage. However, this small difference might have reduced the thermal stress to a level where differences in responses were less pronounced, suggesting that more severe consequences might be observed at higher temperatures [43,45]. This difference also suggests that the fertility loss reported between 27 and 29°C by Zwoinska *et al*. [43] may in fact be an even sharper drop between 28 and 29°C.

In addition to variation in these thermal tolerance lines, Rolandi *et al.* [82] found a window between 40.1 and 41.5°C for the adult critical temperature limit (CT_max_) in the DGRP lines. This study reported that climatic records from Raleigh, where these DGRP lines were collected, showed that from 1980 to 2005, only 10 days exceeded this CT_max_. However, future projections (2045–2070) suggest a dramatic increase to 243 days of extremely high temperatures above the CT_max_ [82]. Thus, heatwaves are expected to become common and frequent in nature in the coming years. Our finding indicates that, regardless of genetic backgrounds, these populations are likely to experience heatwaves across multiple life stages, leading to severe fitness consequences and potential population declines due to prolonged and intense exposure to extreme temperatures that can be compounded across life stages.

## 5. Conclusion

In conclusion, our study demonstrates the cumulative effects of multiple heat stress events on male fitness and its recovery dynamics. The reduced fitness, persisting even after a recovery period, suggests that increasingly frequent heatwaves may contribute to population declines and loss of genetic diversity. Understanding the full scope of heat stress effects, however, requires examining sex-specific responses to heat stress, the impact of life-stage exposure and compounding effects across different stages, and the length, frequency and severity of heat exposure [14,32,54]. Moreover, heat stress effects may be compounded when combined with other environmental challenges [83,84]. Collectively, these challenges may significantly impact biodiversity loss and ecosystem dynamics by altering species interactions and community structure [1,85–87]. Thus, while our findings contribute to a broader understanding of the lasting consequences of heat stress for male reproductive health, further research is needed to predict the consequences of heat stress on population fitness dynamics, along with the underlying the mechanisms of recovery and thermal tolerance.

## Data Availability

Data accessibility is provided at [88]. Supplementary material is available online [89].
